# Emergence of Criticality in the Transportation Passenger Flow: Scaling and Renormalization in the Seoul Bus System

**DOI:** 10.1371/journal.pone.0089980

**Published:** 2014-03-05

**Authors:** Segun Goh, Keumsook Lee, MooYoung Choi, Jean-Yves Fortin

**Affiliations:** 1 Department of Physics and Astronomy and Center for Theoretical Physics, Seoul National University, Seoul, Korea; 2 Department of Geography, Sungshin Women's University, Seoul, Korea; 3 CNRS, Institut Jean Lamour, Département de Physique de la Matière et des Matériaux, UMR 7198, Vandoeuvre-les-Nancy, France; National Research & Technology Council, Argentina

## Abstract

Social systems have recently attracted much attention, with attempts to understand social behavior with the aid of statistical mechanics applied to complex systems. Collective properties of such systems emerge from couplings between components, for example, individual persons, transportation nodes such as airports or subway stations, and administrative districts. Among various collective properties, criticality is known as a characteristic property of a complex system, which helps the systems to respond flexibly to external perturbations. This work considers the criticality of the urban transportation system entailed in the massive smart card data on the Seoul transportation network. Analyzing the passenger flow on the Seoul bus system during one week, we find explicit power-law correlations in the system, that is, power-law behavior of the strength correlation function of bus stops and verify scale invariance of the strength fluctuations. Such criticality is probed by means of the scaling and renormalization analysis of the modified gravity model applied to the system. Here a group of nearby (bare) bus stops are transformed into a (renormalized) “block stop” and the scaling relations of the network density turn out to be closely related to the fractal dimensions of the system, revealing the underlying structure. Specifically, the resulting renormalized values of the gravity exponent and of the Hill coefficient give a good description of the Seoul bus system: The former measures the characteristic dimensionality of the network whereas the latter reflects the coupling between distinct transportation modes. It is thus demonstrated that such ideas of physics as scaling and renormalization can be applied successfully to social phenomena exemplified by the passenger flow.

## Introduction

Recently, there is much interest in applying concepts of statistical mechanics to social systems. For example, such concepts as the phase transition [1], random walk [2]–[4] or phase synchrony [5] were used to understand collective properties, while social networks [6]–[8] provide a simple and convenient description of social systems. The collective properties and associated complexity of the systems in general emerge from the couplings between components, e.g., individual persons [3], transportation points such as airports [9] or subway stations [10], and administrative districts [5], [11]. Among them, criticality is one of the most attractive features in complex systems including social systems.

Hitherto, many of the studies devoted to the emergence of the criticality in complex systems [12]–[17] have been interpreted in terms of self-organized criticality [18] and scale-free power-law distributions have been regarded as the indicator of the criticality at which the typical length scale disappears. However, rather a simple stochastic process can give rise to the power-law distribution [19], [20] and the divergence of the correlation length, namely, the power-law behavior of the correlation function rather than others is crucial in the criticality. Furthermore, in view of the fact that the renormalization group theory of phase transitions provide a profound theoretical framework, validating the scaling relations and universality (see, e.g., [21], [22]), it is desirable to clarify such concepts as the thermodynamic limit, thermal fluctuations and correlations, dimensionality of the system, or relevant coupling between the components, in verifying the criticality based on the theoretical criteria. Unfortunately, however, this task still remains unaccomplished for most of complex systems, due to the limit of the data quality. Notwithstanding this difficulty, there have been attempts to resolve differences between the theory and real data by applying methods of statistical mechanics [15]–[17].

Here the urban transportation system is of great interest as a complex social system, because couplings in the system are accompanied by the movements of people. In recognition of the fact that a majority of people reside in the urban area in this modern age and most of urban flows are realized through the mass transportation system such as the subway or the bus network, the study of the urban transportation system should obviously be essential for probing principles of social systems. Further, recent advances of the smart card technology enable us to gather the information on individual trips and to analyze direct measurement of human movements. In this direction, the Seoul subway system was studied through the smart card data [23]–[25].

In this paper, we consider the Seoul bus system and analyze the smart card data for the passenger flow. The smart card (called the *T-money card* in Seoul) data contain detailed information of each trip including the departure/arrival bus stop and time as well as the bus route taken by the passenger, and thus provide a direct measure on the urban transportation flow. The whole data are collected and managed by the government of Seoul City, and not open to public. With special permission, we have had access to the one week (10–16 April, 2011) data (with personal identification removed) and analyzed the obtained data in detail. It is observed that correlations between numbers of passengers using given bus stops indeed display power-law behavior, which manifests the criticality emerging from couplings between passenger flows. Further, equilibrium fluctuations and the system dimensionality are also clarified. To probe the criticality in more detail, we propose appropriate renormalization processes and perform finite-size scaling on the modified gravity model implementing the couplings between bus stops. The resulting model with the renormalized parameters is shown to give a good description of the Seoul bus system and it is thus demonstrated that statistical mechanics is successfully applicable to the urban transportation flow.

## Results and Discussion

### Seoul Bus System: Criticality and Renormalization

**Figure 1:**
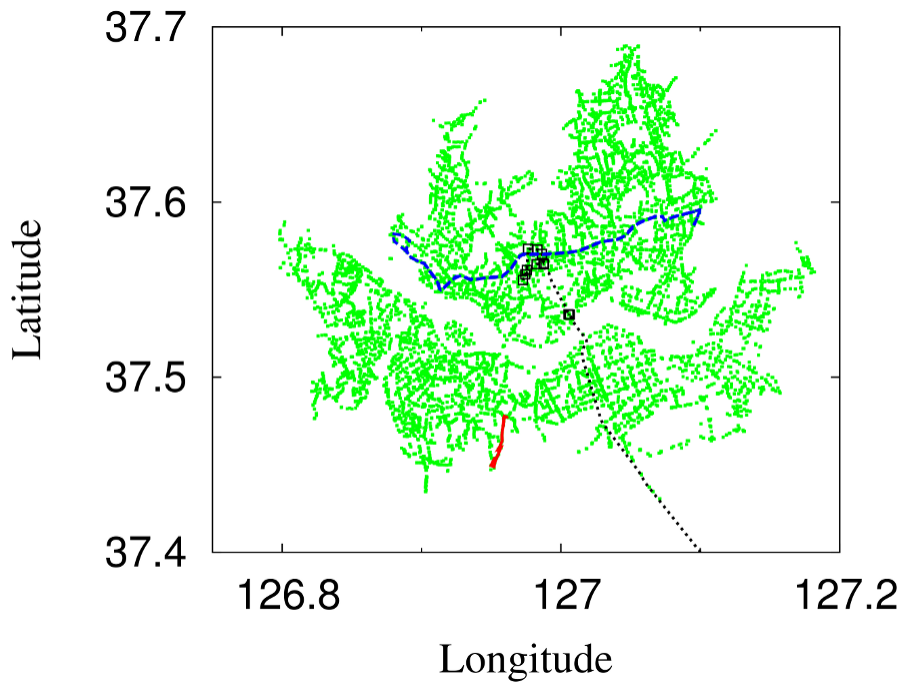
Distribution map of bus stops, together with typical service routes of three characteristic types, in the Seoul bus network. As shown, routes have various service path lengths. A standard city bus route is depicted by the (blue) dashed line. A local shuttle bus route is plotted by the short (red) solid line; the (black) dotted line represents the commuter bus route connecting directly the central business district of Seoul and the satellite city Bundang. Square symbols mark the locations of the bus stops which the commuter bus route serves.

Let us first describe the basic characteristics of the Seoul bus system, which is a major transportation mode in the Metropolitan Seoul with more than 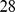
% of the modal share in 2010 [26]. As of April, 2011, there were 595 bus service routes and 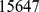 bus stops in total, among which 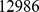 stops were located in Seoul (see Fig. 1). More than six million transactions or completed passenger trips were made on each weekday of the given week in April, 2011. Here we consider the bus network which consists of bus stops serving as nodes and origin-destination pairs of stops as links between nodes.

**Figure 2:**
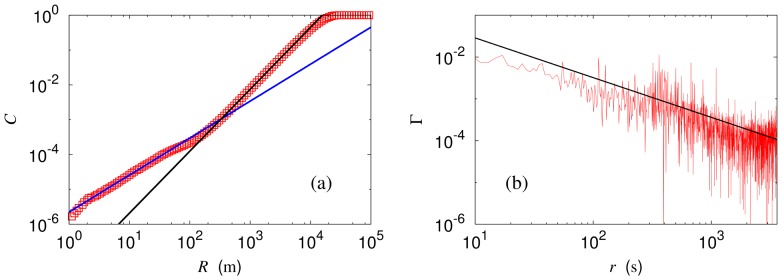
Correlation sum and correlation function. (a) Correlation sum 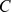 of bus stops versus distance 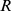 on the logarithmic scale. Also drawn are lines of slope 1.06 and slope 1.78, which correspond to the geographical dimension 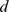 depending on the length scale. (b) Strength correlation function 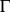 versus time distance 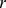 on the logarithmic scale. For comparison, a straight line of slope 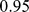 is also shown.

We begin with the dimension of the system, which can be defined according to the distribution of the nodes. Considering the geographical locations and the corresponding spatial distribution of bus stops in the two-dimensional space, we probe the correlation sum of bus stops, i.e., the number of bus stops growing with the (physical) distance from given stop, which gives the correlation dimension of bus stops [27]. The result is plotted in Fig. 2(a). The dimension 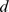 of the network is then close to unity (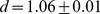) at distances shorter than 200 m, reflecting the linearity of each bus route. At distances between 200 m and 10 km, we obtain the value 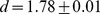. Because the bus stops do not cover the ground compactly due to geographical constraints, this value, less than two, seems reasonable. Further, it is of interest that this also accords with the fractal dimension estimated from the urbanized area distribution [12], [28]. On the other hand, as the length scale reduces below 200 m, the distribution changes strikingly, yielding strong finite-size effects. We also compute the correlation dimension 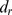 of each bus service route directly from the bus route path, by means of the automatic plateau extraction algorithm [29]. Some bus route paths are not long enough to detect the plateau with given flatness 0.5. Disregarding those, we use 300 bus service routes among 595 routes to estimate successfully the correlation dimension as 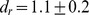, which confirms the origin of the finite-size effects.

On the other hand, the bus network of Seoul is rather sparse in the following sense: Whereas the bus system is embedded on the two-dimensional ground, passenger flows are carried by bus service routes which are specified by roughly one-dimensional arrays of bus stops. The resulting sparse nature of the bus network is conveniently described by the network density defined as the active fraction of all possible links between active nodes:
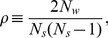
(1)where 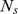 and 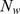 are the total numbers of active nodes and of active links. By active nodes and active links, we mean bus stops used by nonzero numbers of passengers and origin-destination pairs of bus stops carrying nonzero passenger flows, respectively. Data for these characteristics of the Seoul bus network are summarized in Table 1. Note that the network shares essentially the same behavior on weekdays.

**Table 1:** Statistical properties of the Seoul bus network.

	Transaction	No-transfer	Transfer-subway	*N_s_*	*N_w_*	*ρ*
Monday	6,118,063	2,465,802	1,673,396	15,558	388,611	0.00321
Tuesday	6,197,066	2,506,855	1,688,808	15,553	390,277	0.00323
Wednesday	6,244,874	2,535,658	1,693,175	15,555	390,699	0.00323
Thursday	6,190,614	2,515,078	1,684,666	15,555	390,058	0.00322
Friday	6,160,585	2,533,688	1,653,979	15,552	389,362	0.00322
Saturday	5,349,482	2,438,953	1,266,950	15,512	377,511	0.00314
Sunday	3,549,359	1,669,990	803,016	15,439	335,420	0.00281

The total number of transactions regardless of transfers, the number of transactions without transfers to other bus routes or the subway, and the number of transactions with transfers to the subway are given on each day. Also shown are the number 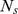 of active nodes and the number 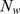 of active links as well as the network density 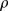.

In general, different bus stops and routes are coupled crucially to each other, as revealed by the fraction of transfers occupying more than half the total transactions. This gives rise to interesting collective behaviors of the passenger flows, manifested by correlations in the system. Specifically, we focus on the strength 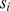 of bus stop 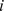, which corresponds to the number of passengers using the bus stop, and consider the strength correlation function. In view of the strength taking very different values depending on the bus stop and the multiplicative Yule type process in transportation systems [24], [25], we consider the normalized strength 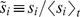 in probing correlations, where 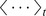 represents the time average over five weekdays. Here data on single weekdays correspond to microscopic states, and the average taken over them describes the macroscopic state. Accordingly, the correlation function of normalized strengths is defined to be
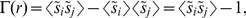
(2)where stops 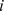 and 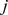 are separated by time distance 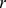 on a bus service route and 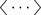 represents the spatial average taken over all stops as well as the time average over five weekdays. Here the time distance between two bus stops corresponds to the time required for traveling between the two stops. More specifically, it is defined to be the average travel time taken by passengers, as extracted from the smart card data. Because the spatial distance is apt to be distorted by geographical factors such as topography or traffic congestion [30], the time distance serves as a more appropriate measure capturing the actual economic cost or the social distance experienced by the passengers. In picking out bus stop pairs for given time distance, we allow a tolerance of two seconds and compute the correlation function for the Seoul bus network with 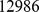 bus stops, to obtain the power-law behavior 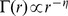 with exponent 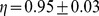, as shown in Fig. 2(b). Such power-law correlations manifest emergence of criticality [22], which is characterized by scale invariance, and conveniently probed by means of scaling and renormalization. Renormalization was applied both to model networks and to networks constructed from data [31], [32], and corresponding flows and fixed points were examined [33]. Note that the bus network is basically a spatial network rather than a topological one: Couplings between bus stops depending on the physical distance are of importance, making it necessary to perform geographical scaling [34].

In the following, we develop the renormalization process appropriate for the spatially embedded network, and apply it to the bus network. The spirit of the renormalization is similar to that of Monte Carlo renormalization [35]–[37] and we treat the transaction data to reveal the flows and fixed points of the couplings. We consider two different procedures of renormalization, “box renormalization” and “node renormalization”, and compare the results, to demonstrate their applicability.

**Figure 3:**
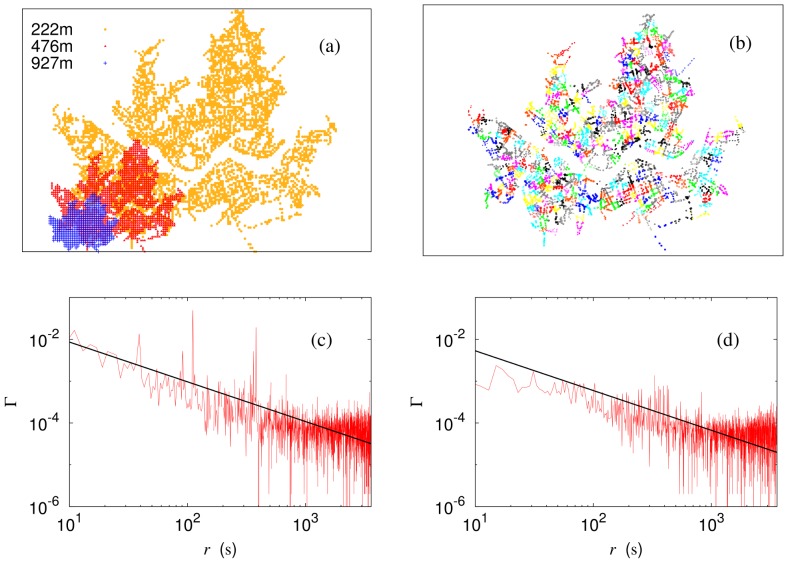
Renormalization and correlation function in the two schemes. (a) Box renormalized systems for block size 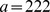 m, 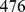 m, and 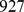 m. (b) Node renormalized system for 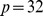 or effective block size 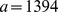 m. Nodes of the same color and symbol represents bus stops composing a single block stop. (c) Correlation function in the box renormalized system for 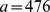 m, together with the straight line of slope 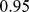. (d) Correlation function in the node renormalized system for 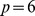 or 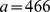 m, together with the straight line of slope 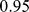.

In the case of box renormalization, we partition the whole area of Seoul into square boxes of given size 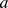, similarly to the renormalization process for the topological network [32]. The difference lies in the concept of the distance: It is defined geographically for the spatially embedded network, whereas the chemical distance is used for the topological renormalization of the network. Those bus stops within each box are then considered to form a “block stop”, thus reducing “degrees of freedom”. This is followed by scale transformation, the details of which are described as follows: (1) Partition the system into square boxes of linear size or “lattice constant” 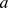. (2) Obtain the number of passengers using any bus stops in each box, which gives the strength of the corresponding block stop. Accordingly, we obtain the passenger flows between block stops as well. (3) Compute the relevant parameters in the renormalized system consisting of block stops. (4) Increase the block size 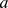 and repeat the above procedure. Here the increase in the block size is followed by the scale transformation under which the spatial length scale shrinks by the same proportion. Namely, the linear size 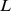 of the system is reduced, set inversely proportional to the block size: 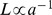. To be specific, we choose 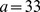 m, corresponding to 0.0003 degree of latitude, as the fundamental size and perform renormalization in which the block size is increased by 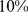 at each stage, ranging from 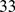 m to 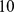 km. Namely, the block size at the 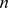
th stage is given by 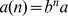 with the scale factor 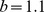. The renormalized systems with block size 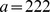 m, 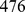 m, and 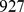 m are exhibited in Fig. 3(a).

In node renormalization, nearby bus stops are combined to form a block stop. We partition all the bus stops into groups, each consisting of 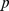 neighboring bus stops. This process is repeated as 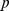 is increased from unity. The effective block size (lattice constant) 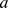 is determined in the following way: First, the center of a block stop is given by the average location of bus stops consisting the block stop. Then the average block radius is defined as the average of the root-mean-square distance of the constituent stops from the center: 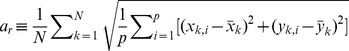, where 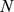 is the total number of block stops, 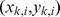 is the location of the 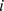
th stop of the 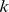
th block stop and 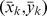 is the center of the 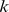
th block stop. Finally, the average block radius multiplied by an appropriate scale factor 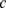 is defined to be the effective block size: 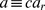. The scale factor 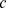 would be two if all block stops formed close packing of perfect circles; otherwise 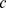 is greater than two. Here we fix 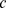 to have the same network density profile data in the two renormalization schemes collapse onto each other (see Eq. (5) below), which yields 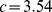. Technically, we employ simulated annealing, with the average block radius as the cost function to minimize, and obtain the optimal partitioning into groups of 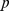 nearest neighboring stops for 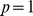 to 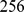. To achieve this, we first carry out the greedy algorithm, the result of which is adopted as the initial configuration (block stop distribution). While cooling the temperature algebraically toward “zero temperature”, where the acceptance probability is about 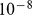, we perform 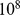 Monte-Carlo steps at each temperature. It turns out that the average block radius is about 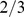 of that obtained via the greedy algorithm and there remain no pathological block stops consisting of spatially separated groups of bus stops. As an example, the renormalized system for 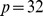, corresponding to 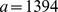 m is shown in Fig. 3(b).

In both renormalization schemes, the distance between block stops, i.e., the lattice constant 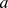 would be made unchanged, if the system gets properly shrunk under the scale transformation, as shown in Fig. 3(a). However, since we deal with the time distance rather than spatial one, we save the trouble of taking the spatial shrinkage step and describe the system with the original size to preserve its geographical properties. In fact this is closely related with how to increase the degrees of freedom and to reach the thermodynamic limit of the system. Usually, the thermodynamic limit is attained by increasing both the degrees of freedom and the system size with the density being fixed. For the bus network system, however, increasing the spatial size of the city would also increase characteristic time scales of urban transportation modes and thus change the relations between them. Therefore, it is more convenient for the transportation system to reach the thermodynamic limit by increasing the density, namely, by increasing the number of bus stops while keeping the spatial size of the city unchanged. In this view, the finite-size effects associated with the lack of a sufficiently large number of bus stops are attributed to the finite density of bus stops rather than the finite size of the area. The thermodynamic limit 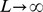 with the linear size 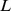 in units of 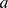 may thus be reached by increasing the density, i.e., by taking the limit 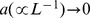. This is just the reverse of the scale transformation in the renormalization process where 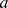 is increased by the construction of block stops. As the renormalization proceeds, the numbers 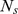 and 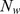 of active nodes and links also keep decreasing because some neighboring stops are replaced by single block stops. We count the number of block stops of nonzero strength and the number of renormalized links with nonzero weight on each renormalization stage.

To check the criticality of the renormalized system, we compute the correlation function given by Eq. (2) and show the behavior in Fig. 3(c) and (d). Data in the range between 50 s and 1000 s are used for fitting, which cover 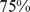 of the total 28,617,196 passenger transactions. Power-law behaviors are observed with exponent 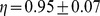 and 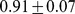 in the box and node renormalization schemes, respectively, which agree with the “bare” (unrenormalized) behavior in Fig. 2(b) within error bars; this confirms the criticality of the system.

**Figure 4:**
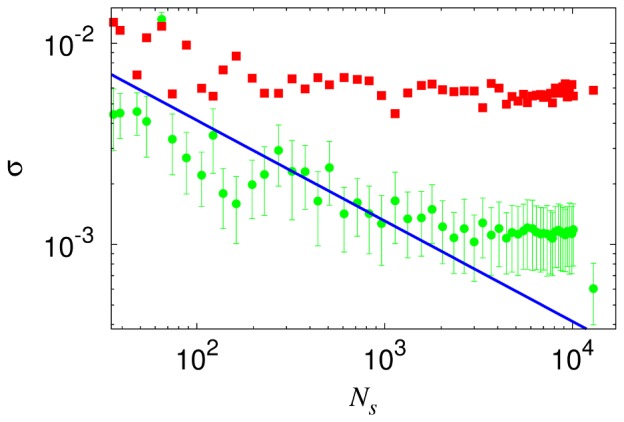
Anomalous scaling of the temporal fluctuations. Red squares represent rms fluctuations of the original data; green circles show the results obtained from 100 randomly shuffled data, with the error bars corresponding to one standard deviation. The blue solid line, having the slope 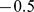, corresponds to 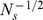. The saturation for 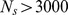 is due to incomplete randomization.

For additional verification of the criticality, we also examine the scaling behavior of temporal fluctuations, following the idea in Refs. [3], [6]. At the critical point, fluctuations should be scale invariant, emerging at all scales. To consider temporal fluctuations, we first define the mean (normalized) strength of the bus system
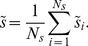
(3)and its standard deviation
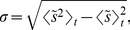
(4)which measures the root-mean-square (rms) temporal fluctuations of the mean strengths. If there were no correlations, the rms fluctuations should decay with the size (number of nodes) as 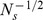, simply according to the law of large numbers. In reality, Fig. 4 shows that the rms fluctuations of the original data (plotted with red squares) remain essentially unchanged as the size 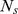 is varied; such anomalous scaling manifests scale invariance of the fluctuations or criticality. For comparison, we shuffle randomly the temporal sequences of the strengths of bus stops, breaking correlations between them, and construct renormalized systems from the shuffled data. The corresponding rms fluctuations of shuffled data indeed decay with the system size as 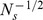 (see the data with green circles in Fig. 4). These results indicate that the anomalous scaling has its origin in strong correlations present in the original data, confirming the criticality in the system.

Here one should be careful about renormalization with large block size, since there is a cutoff originating from sparseness of the network. To probe this, we examine the scaling behavior of the network density defined by Eq. (1). In the system of geographical dimension 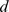 and linear size 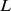, the number of nodes scales as the 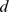-dimensional volume: 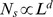. On the other hand, if each bus route is described by a 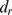-dimensional array of bus stops, the number of bus stops on a route scales as 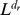, leading the number of links on each route to scale as 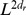. Because each bus route shares this scaling property, the total number of links in the whole bus system also scales as 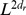. Accordingly, we have the network density
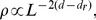
(5)which decreases as the system size 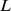 grows (since 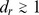 and 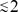).

Heretofore, we have assumed that the overlap of bus routes does not change significantly as the box size is increased. When the size 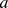 becomes larger than certain threshold 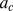, however, the overlap increases significantly with renormalization, making Eq. (5) fail for 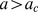. In fact, there exist overlaps of bus routes even in the bare bus system: Most of bus routes share their paths with some other routes and on average 2.35 routes serve each bus stop. As renormalization proceeds, i.e., as 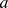 is increased and more stops merge into single block stops, some bus routes which do not serve the same bus stop in the bare system serve the same block stop in the renormalized system; this leads the overlap to increase. Here the threshold 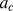, which may be interpreted as the typical distance between nearby bus routes without overlap, turns out to be around 1 km. Note that development of such additional overlaps of bus routes affects the sparseness of the network. In general the bare network is sparse, which implies that coupling is essentially short ranged: Despite that bus routes can link those bus stops very far from each other, there is limitation arising from the strong anisotropy of each route and each bus stop can couple to only a small fraction of bus stops. As 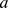 or 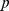 is increased, however, the short-range nature of the coupling is lifted and the increase in the overlap of bus routes drives the network finally toward the fully connected limit; this affects the universality class and critical behavior of the system. Therefore, renormalization, so as to preserve the short-range nature of the coupling, should not proceed indefinitely.

**Figure 5:**
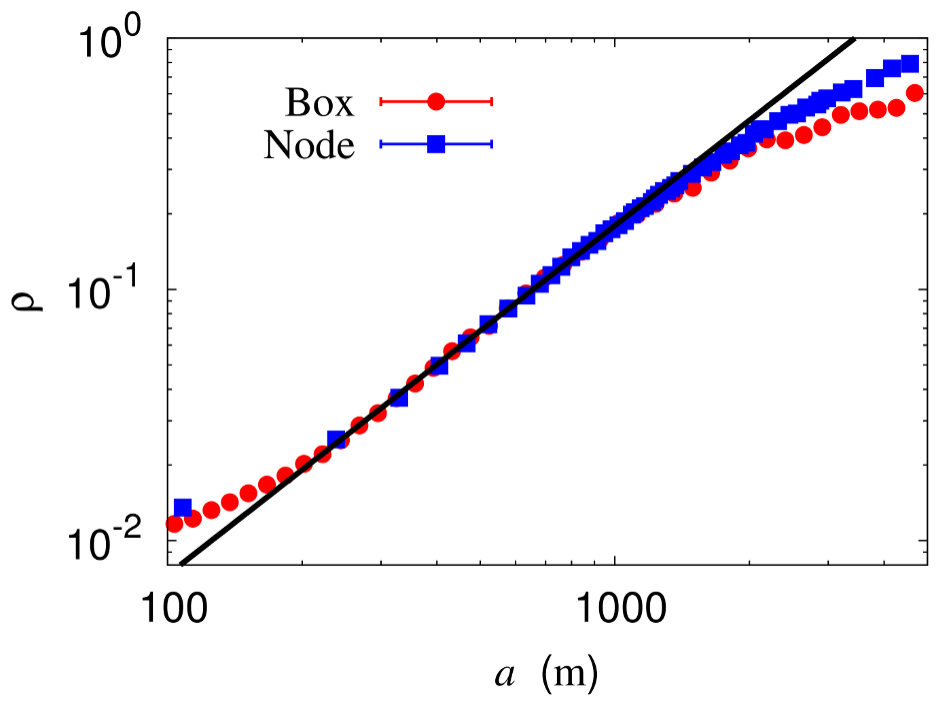
Network density 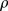 versus (effective) block size 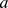 on the logarithmic scale. Red circles and blue squares represent the data in the box and node renormalization schemes, respectively. Observed is algebraic growth with exponent around 1.4, as plotted by solid lines fitting the data in the range 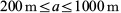.

Figure 5 shows how the network density varies with the block size, i.e., flow of the network density under renormalization [38]. It is of interest that the densities in both renormalization schemes collapse onto each other, which has fixed the scale factor 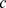 in node renormalization. Observed is the power-law growth with the block size: 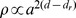, which is just Eq. (5) since 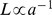. The exponent takes the value 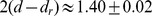 in both renormalization schemes, until the block size 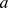 reaches the cutoff 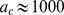 m. This result, together with the geographical dimension of the network 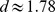 obtained from the scaling behavior of the correlation sum, leads to the dimension of the bus service route 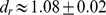, which appears consistent with the correlation dimension of bus stops at short distances (below 200 m) and/or the dimension obtained directly from each bus service route. Whereas deviations from the power-law behavior for 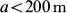 reflect the finite-size effects, those emerging for 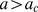 indicate that the nature of the coupling is altered. Namely, the network becomes no longer sparse due to the substantial overlaps between bus routes and as a result, the universality may change, disallowing renormalization beyond 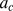.

### Modified Gravity Model

To illustrate the utility of the renormalization theory, we apply it to the gravity model, which provides a prototypical description of passenger flows in transportation networks. The conventional gravity model may suffer from oversimplifications. Specifically, limitations due to the geographical non-uniformity have been addressed, together with possible modifications to overcome the limitations [39], [40]. In the case of the intra-urban flow, on the other hand, transportation facilities are among the main factors which reflect the non-uniformity originated from the modularized structures and distinctive functional connections between them. In this direction, the modified gravity model was proposed [25], based on non-transfer trips; this makes it possible to probe the bare coupling between the nodes. Note that there in general exists cooperation or competition among different transportation modes, e.g., walk, bus, or subway, which gives rise to coupling between corresponding transportation networks. Such coupling can be taken care of by introducing modulation of the gravity model by the Hill function. This leads to the modified gravity model:
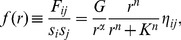
(6)where 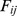 is the passenger flow between nodes 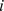 and 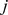 and 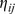 describes fluctuations. The reduced flow 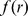 thus depends only on the time distance 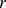 between nodes 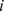 and 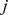; 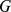 is the overall proportionality constant and 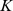 is the time constant separating the long- and short-distance regimes. In this model, parameters of relevance are the gravity exponent 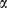 and the Hill coefficient 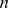: The former governs the long-distance behavior whereas the latter measures the number of coupled transportation modes. In the case of the Seoul subway system of the year 2005, we observed that 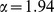, 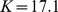 min and 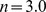, an integer within error bars. In addition, fluctuations 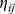 were found to follow a log-normal distribution quite accurately, implying the Yule-type nature of the transportation system [25].

**Figure 6:**
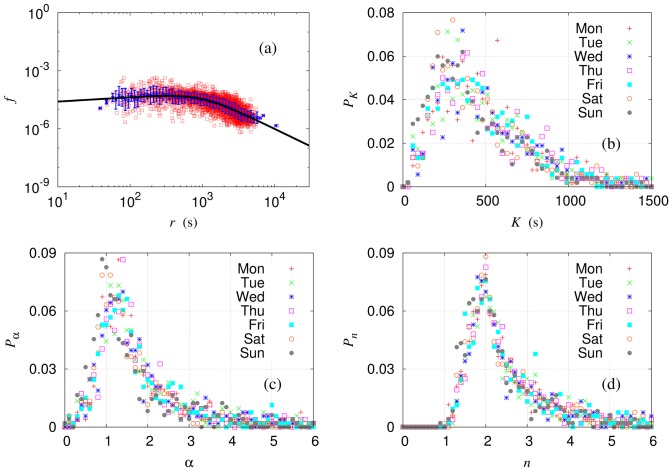
Modified gravity model applied to each service route. Symbols represent data points and solid lines describe model results, with error bars estimated by standard deviations. (a) Reduced flow 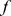 versus time distance 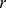 on the logarithmic scale, for route No. 271 (which is a typical standard city bus route plotted in Fig. 1 of the main paper) on Monday, when there were 36569 transactions in total. The least-squares fitting to the model gives the parameters 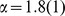, 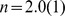, and 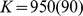 s. In (b), (c), and (d), daily distributions of the time constant 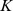, gravity exponent 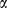, and Hill coefficient 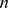, respectively, are plotted for 595 bus service routes.

We first apply the modified gravity model to each of the 595 routes in the Seoul bus network. Note that some routes serve bus stops in satellite cities which are not plotted in Fig. 1. For most routes, the model gives a good description of the passenger flows at short- and long-time distances with appropriate crossovers between them, see Fig. 6(a). On the other hand, the model does not apply well to those routes with too few bus stops, for which insufficient degrees of freedom disallow least-squares fitting with reasonable values of the time constant 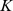; a majority of commuter bus routes and local shuttle bus routes belong to this category. Such routes, accounting for 11.3% of the 595 routes on weekdays, are excluded in further analysis.

Despite the good description for each individual route, resulting model parameters vary substantially with the route and follow the distributions plotted in Figs. 6(b), (c), and (d). It appears that the role of a bus route is influenced much by the geographical environment such as nearby subway stations as well as such characteristics as the total number of bus stops and the total path length of the route. This contrasts sharply with the subway system which displays universality among different lines, depending only on topology [25].

**Figure 7:**
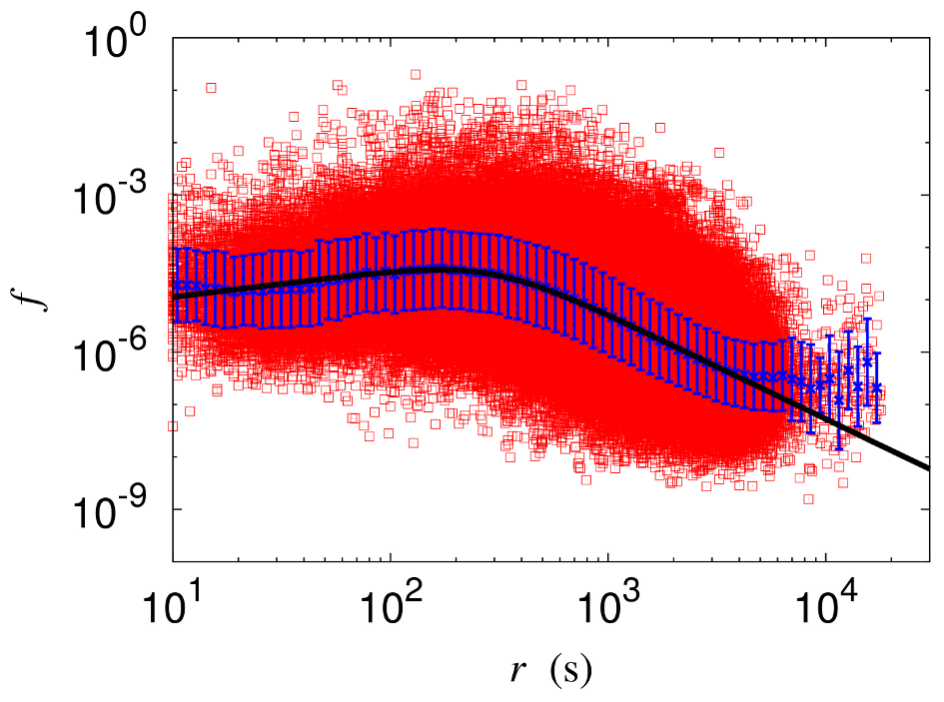
Modified gravity model applied to the whole bus network. Symbols represent data points and solid lines describe model results, with error bars estimated by standard deviations. Reduced flow 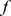 versus time distance 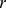 on the logarithmic scale, for the whole bus network on Monday. Deviations at distances larger than an hour (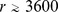 s) have the origin in boundary effects.

The modified gravity model is then applied to the whole bus system in Seoul. Namely, we obtain the reduced flows on all links between all the bus stops, as shown for Monday in Fig. 7. The corresponding values of the parameters, obtained via least-squares fitting, are summarized in Table 2. Note also that the Hill coefficient takes non-integer values, which appears unphysical. This can be remedied by carrying out scaling analysis, as discussed in the next section.

**Table 2:** Parameters of the modified gravity model.

	Bare system				Box renormalization		Node renormalization	
	*α*	*n*	*K* (s)	Reduced *χ* ^2^	*α*	*n*	*α*	*n*
Monday	2.00(1)	2.50(1)	291(4)	2.80	1.66(2)	2.00(2)	1.69(4)	2.06(4)
Tuesday	2.00(1)	2.51(1)	292(4)	2.78	1.67(2)	1.97(2)	1.73(2)	2.03(5)
Wednesday	2.01(1)	2.53(1)	292(4)	2.78	1.68(2)	2.02(2)	1.71(3)	2.03(6)
Thursday	2.00(1)	2.52(1)	292(4)	2.79	1.68(2)	2.01(2)	1.74(2)	2.06(4)
Friday	2.00(1)	2.51(1)	292(4)	2.78	1.68(2)	2.03(3)	1.74(2)	2.05(5)
Saturday	1.94(1)	2.48(1)	282(4)	2.85	1.61(2)	1.94(2)	1.68(2)	1.96(6)
Sunday	1.87(1)	2.39(1)	263(4)	2.89	1.52(2)	1.87(2)	1.61(2)	1.92(5)

Gravity exponent 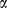 and Hill coefficient 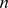 as well as time constant 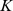 for the whole bus network, together with their renormalized values are given on each day of the week. Fitting errors for the whole bus network are also shown. It is observed that the renormalized values obtained from box renormalization and from node renormalization are essentially the same. As discussed in the text, the renormalized gravity exponent measures the characteristic dimensionality of the bus network whereas the renormalized Hill coefficient takes the integer value (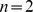) within error bars.

### Scaling Analysis

**Figure 8:**
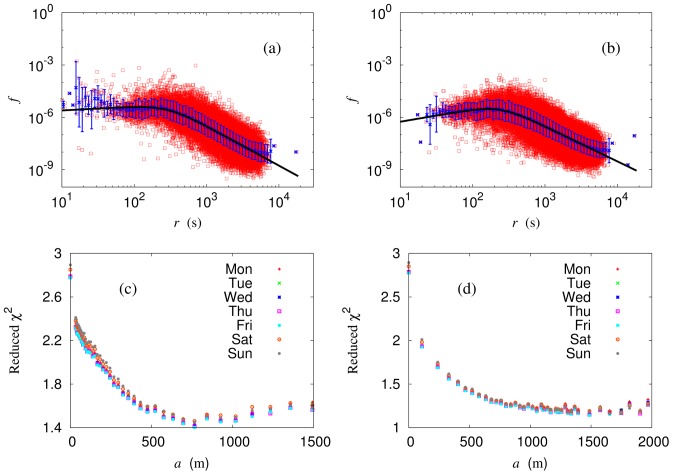
Modified gravity model applied to the renormalized system. Reduced flow 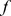 versus time distance 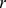, obtained from the same data as Fig. 7: (a) box renormalization with 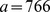 m and (b) node renormalization with 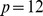. Reduced 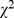 versus (effective) block size 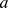: (c) box renormalization and (d) node renormalization. Data for the renormalized system fit better and better as 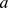 is increased up to 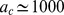 m.

Making use of the criticality, we perform renormalization and apply the modified gravity model to the renormalized system, as shown in Fig. 8(a) for box renormalization and Fig. 8(b) for node renormalization. It is remarkable that the renormalized system fits excellently into the modified gravity model (compared with the bare system); In particular, the description becomes more and more precise as renormalization proceeds up to the cutoff 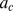 illustrated in Fig. 8(c) and Fig. 8(d), exhibiting reduced 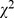 versus the block size 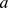 in the renormalized system. Note that the cutoff 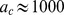 m specifies precisely the scaling regime (see Fig. 5). Now we can obtain the renormalized values of the modified gravity model parameters by taking the thermodynamic limit, i.e., extrapolating the data points in the scaling regime to the limit 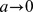.

**Figure 9:**
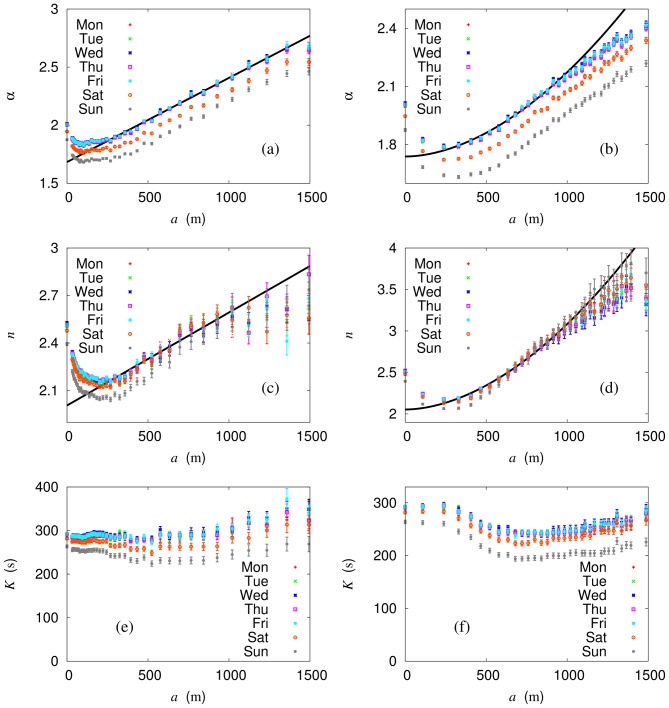
Scaling analysis of the Seoul bus network. The gravity exponent 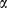, Hill coefficient 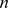, and time constant 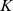 versus (effective) block size 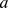 for box renormalization [(a), (c), and (e) on the left column] and for node renormalization [(b), (d), and (f) on the right column] are shown together with least-squares fitting lines in the scaling region. Extrapolation to the limit 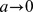 leads to the renormalized values summarized in Table 2.

Values of the model parameters, obtained in the process of renormalization, are plotted versus the block size in Fig. 9, both for weekdays and for the weekend. As expected, the time constant 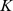 remains essentially unchanged in the scaling region. On the other hand, the gravity exponent 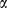 and the Hill coefficient 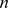 are shown to decrease algebraically as the block size reduces (or as the system size grows). For 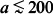 m, as expected in Fig. 2(a), this scaling behavior is concealed by finite-size effects and should be extrapolated; note that data points at 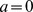 represent the results of the bare system summarized in Table 2.

Choosing the scaling region for the network density (see Fig. 5) and fitting the data in that region, i.e., for the block size in the range 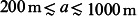, we obtain essentially the same values of the gravity exponent and the Hill coefficient for both renormalization schemes. The resulting renormalized values of the gravity exponent 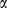 and the Hill coefficient 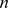 are summarized in Table 2. It is of interest to compare these results with the non-renormalized values which suffer from finite-size effects, making it necessary to perform renormalization to obtain physical values. In particular, upon renormalization, the Hill coefficient appears to take the integer value (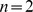) within error bars. It is thus reasonable to conclude that 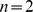, which implies that there is another transportation mode coupled to the bus at short distances, perhaps walking as an alternative to the bus.

Meanwhile, the gravity exponent 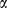, measuring the dependence of passenger flows on the (time) distance, is related closely to the dimension of the system, defined appropriately. At long distances, one may disregard the modification by walking and apply Gauss' law to passenger flows regarded as fluxes. In the system of appropriate dimension 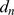, this leads to 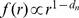, which implies the relation 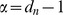.

**Figure 10:**
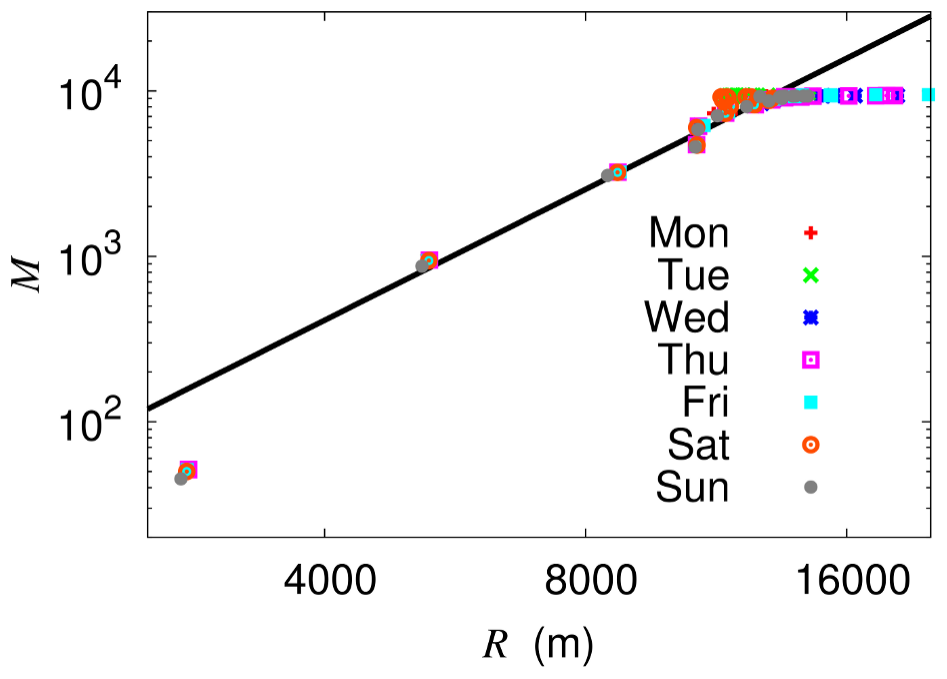
Number 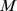 of linked nodes versus distance 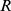, together with the line of slope 2.63. This gives the network dimension of the bus system, considering both geographical and topological characters.

The geographical dimension 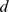, which we have considered, describes the spatial distribution of bus stops and manifests the origin of finite-size effects in the system. This dimension, however, does not serve the purpose in the bus network embedded on the two-dimensional surface, where passenger flows exist on links and depend on the (time) distance. Instead, the network dimension 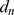 taking into account both geographical and topological characters should be considered. We thus examine how passenger flows on links vary with the time distance, adopting the method for the spatially embedded network [41], and plot the results in Fig. 10. There the network dimension is given by 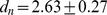, which indeed agrees with 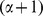 within error bars. We can finally conclude that the individual feels the force with the exponent 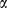 in the network embedded in 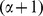 dimensional space. This shows that the functional connectivity, i.e., the linkage via bus service routes, added to the geographically embedded bus stops, explains the gravity exponent successfully, and suggests the possibility that in a network of given geographical and topological structure and node strengths, passenger flows between nodes can be obtained from the gravity model.

## Conclusions

Analyzing the smart card data during a week, we have studied the passenger flow on the Seoul bus network system, to find power-law correlations, characterized by algebraic behavior of the strength correlation function of bus stops. By analogy with statistical mechanics, daily variations of strengths have been treated as thermal fluctuations and such criticality has been probed by means of the scaling and renormalization analysis of the modified gravity model applied to the system, which has revealed the underlying structure of the system. In addition the dimensions of the system have been clarified and their roles in the density scaling been examined. It has been demonstrated that the resulting renormalized values of the gravity exponent and of the Hill coefficient give a good description of the Seoul bus network: The former measures the characteristic dimensionality of the network whereas the latter reflects the coupling between distinct transportation modes.

As criticality emerges in this transportation system and renormalization has been carried out, it is tempting to find analogies and correspondences with physical models studied extensively in their critical regimes by means of tools of statistical physics, such as phase transitions in magnets or self organized critical systems. It may also be interesting to isolate the nature of long- or short-range interactions at play, which could explain the emergence of collective effects essential for the criticality in the network. It is obvious that travel behavior is dictating the interactions since passengers do not use bus routes in a random way but want to minimize the time between the origin and destination and to enhance the mobility during peak periods. Here such a local variable as the strength of each bus stop is used as the local order parameter; the bus network is described by the graph at each vertex of which these variables are located. Usual quantities such as correlation functions are then useful in determining the existence of a critical regime, from which typical scaling exponents at large distances are extracted. Unfortunately, it is not obvious how to identify the control parameters corresponding to the temperature or external fields. For instance, one may consider the government policy or the economic and political state as a candidate for the social temperature while the population flow from other cities may serve as the external pressure for a city in the growth or downfall stage. Some (social or natural) regional events may also be regarded as local external fields. Further, local disturbances on some route paths, due to maintenance, road works, or delays, can serve to study response functions. These functions express the way the passenger flow is reorganized at intrinsic time scales, after an external perturbation is imposed. However, the study clarifying relevant control parameters is yet to be made and at this stage it is not conceivable to derive the scaling relations in the prototypical form. Nevertheless, the relation between the network density exponent and the fractal dimension of the system appears promising, for the dimensionality plays a crucial role in the scaling relations.

It also needs further study to reveal detailed nature of the microscopic couplings between bus stops and to identify reduced flows and couplings. Although the reduced flow may not be exactly equivalent to the coupling constant in a physical system, one may speculate that the strengths of stations linked via large flows may tend to order, as spins are aligned in the same direction when the couplings between spins are strong. In this way, the reduced flow can be regarded as the coupling between strengths, and we conclude that the values obtained via scaling analysis are closely related to the renormalized couplings in the Seoul bus network.

Note that criticality or power-law behavior of correlations emerges as a characteristic of the strength distribution across the system. It is natural that the distribution of passengers or populations is a consequence of the couplings between bus stops or regions of the city. As well known, the competition between order attained by couplings and disorder reflecting fluctuations or randomness is crucial for criticality. In the growth and fall of a city, these properties appear to be organized to generate the complex structure of the city. In conclusion, understanding the couplings and fluctuations is essential for establishing theoretical models for transportation systems and cities, which is left for further study.
